# Imaging Measurement of Whole Gut Transit Time in Paediatric and Adult Functional Gastrointestinal Disorders: A Systematic Review and Narrative Synthesis

**DOI:** 10.3390/diagnostics9040221

**Published:** 2019-12-13

**Authors:** Hayfa Sharif, David Devadason, Nichola Abrehart, Rebecca Stevenson, Luca Marciani

**Affiliations:** 1 Nottingham Digestive Diseases Centre, University of Nottingham, Nottingham NG7 2UH, UK; hayfa.sharif@nottingham.ac.uk (H.S.); msznja@exmail.nottingham.ac.uk (N.A.); 2 Amiri Hospital, Ministry of Health, Civil Service Commission, Kuwait City 12025, Kuwait; 3 National Institute for Health Research (NIHR), Nottingham Biomedical Research Centre, Nottingham University Hospitals NHS Trust and University of Nottingham, Nottingham NG7 2UH, UK; 4 Nottingham Children’s Hospital, Nottingham University Hospitals NHS Trust, Nottingham NG7 2UH, UK; David.Devadason@nuh.nhs.uk; 5 Precision Imaging Beacon, University of Nottingham, Nottingham NG7 2UH, UK; mszrhs@exmail.nottingham.ac.uk

**Keywords:** imaging, transit, colon, MRI, constipation, irritable bowel syndrome, dysmotility, small intestinal bacterial overgrowth

## Abstract

Background: functional gastrointestinal disorders (FGID) are common conditions in children and adults, often associated with abnormalities of whole gut transit. Currently, transit tests can be performed using several imaging methods, including tracking of radiopaque markers, gamma scintigraphy with the use of radioisotopes, magnetic tracking methods, tracking of movement of wireless motility capsules, and emerging magnetic resonance imaging (MRI) approaches. Objectives: to review recent literature on diagnostic imaging techniques used to investigate whole gut transit in FGIDs. Methods: a systematic review was carried out. The different techniques are described briefly, with particular emphasis on contemporary literature and new developments, particularly in the field of MRI. Conclusions: emerging MRI capsule marker methods are promising new tools to study whole gut transit in FGIDs.

## 1. Introduction

Functional gastrointestinal disorders (FGID) are some of the most commonly diagnosed conditions in gastroenterology and account for about 40% of all gastroenterology clinical practice [1]. These disorders can be associated with abnormalities of transit and diagnostic testing can be clinically useful to detect abnormalities [2]. 

The Rome IV process provides consensus definitions and diagnostic criteria classifying FGIDs according to the segment of gastrointestinal tract where the condition is believed to originate from [1,3]. Rome IV classifications were also developed for FGIDs observed in children and adolescents [4,5]. Constipation features prominently as a common complaint in multiple FGIDs (according to Rome IV [3]). Three groups of bowel disorders (irritable bowel syndrome with predominant constipation (IBS-C), functional constipation and opioid-induced constipation) are associated with constipation. Small intestinal bacterial overgrowth (SIBO) is another common disorder of the gastrointestinal tract. It reflects excess presence of bacteria in the small bowel, and recent studies suggest SIBO can be associated with altered gut transit [6].

Constipation can be caused by slow transit of chyme within the colon. Different factors can contribute to this, including diet, lifestyle and medications [7]. Constipation can also signal the presence of other underlying disorders such as diabetes, coeliac disease and cancer [8].

A recent review estimated the average prevalence of constipation at 16% worldwide [9]. In the United States, the number of hospital emergency visits linked to constipation increased by 41.5% between 2006 and 2011 [10]. Constipation is thought to be twice as common in women than in men [9]. Women at the postpartum stage (several weeks after pregnancy) are often affected by the condition, with up to 25% of women reporting the incidence of constipation [11]. Constipation rates tend to be substantially higher in the elderly population and reach up to 50–75%.

Most cases of constipation are caused by one of three general mechanisms: disordered and obstructed defecation caused primarily by impaired rectal evacuation, irritable bowel syndrome with constipation, and slow transit constipation [12]. These mechanisms differ substantially, even though the general symptoms of constipation appear very similar. Proper treatment in each case calls for a patient-specific differential diagnosis. This diagnosis remains challenging and about half of all patients who complain of constipation remain unsatisfied with the treatment they receive [12]. 

Slow transit constipation, a reduced intestine motility caused by abnormalities of the enteric nerves, accounts for 15–30% of all constipated patients, with up to 37% of constipated women affected [13]. 

Functional constipation in childhood is very common. Its prevalence has been estimated to be 14%, forming approximately 3% of all hospital paediatric referrals [14,15,16,17,18]. In the majority of paediatric cases, constipation is not linked to specific clinical disorders but rather caused by changes in lifestyle or diet [19]. Slow transit constipation accounts for approximately 10% of all paediatric cases [20]. Managing illness in these children is challenging, and the diagnosis often unclear and based mostly on symptom reports [5,21]. An objective measure of the gut transit time could assist in stratifying the patients and direct early selection of therapy. Gastrointestinal transit time in children is, however, not often assessed using current methods due to the radiation dose involved, which is not suitable for young patients due to their greater sensitivity to radiation than adults.

As the etiology of constipation is multifactorial, the treatment of constipation requires a clear understanding and a differential diagnosis of its clinical causes [22]. Physical examination (abdominal and rectal examinations) and various tests can be performed, including imaging studies of whole gut transit time. Transit time in this context is the time taken by food and chyme to travel through the gastrointestinal tract, thanks to complex propulsive mechanisms. Whole gut transit time (WGTT) is defined as the combination of gastric emptying, small bowel transit time and colonic transit time [23].

The ideal technique for these measurements should be noninvasive and easily applicable for both diagnostic and patient monitoring in the course of therapy. Currently, colonic transit can be performed using several imaging methods, including the use of radiopaque markers, radioisotopes, methods to track magnetic capsules or wireless devices and magnetic resonance imaging (MRI) approaches. 

New MRI-based methods to measure and monitor the gastrointestinal transit are currently emerging, with the possible benefits of the nonionising, cross-sectional imaging that MRI offers, which is particularly suited to the paediatric population. The anatomy can be visualised in detail whilst assessing transit.

## 2. Materials and Methods

A systematic search of the literature was carried out to identify publications describing imaging studies of gastrointestinal transit. The Preferred Reporting Items for Systematic Reviews and Meta-Analyses (PRISMA) guidelines were followed. 

The primary searches were carried out in Web of Science complemented by searches on Medline and Embase engines using keywords relating to gastrointestinal transit and functional gastrointestinal diseases. The search strategy is provided in Supplementary Materials Figure S1. 

Studies were included in this review if they matched the predefined inclusion/exclusion criteria according to the patients, intervention, comparator, outcomes, and study design (PICOS) tool as reported in Table 1.

Duplicates were removed automatically using EndNote reference management software. The included records were then screened by the title, abstract, and full text by two authors (H.S. and L.M.). Once a paper was found to fit the eligibility criteria, its references were, in turn, searched in order to find new papers that were previously missed.

Each included study was assessed for potential risk of bias (for example from the study design and reporting) by two researchers independently (H.S. and R.S.) using the ROBINS-I (Risk Of Bias In Non-Randomized Studies-of Interventions) tool [24,25]. Seven domains of bias were evaluated and the preintervention domains were biased due to confounding and bias in selection of participants into the study. The intervention bias domain was biased in classification of interventions. The postintervention domains were biased due to deviations from intended interventions, bias due to missing data, bias in measurement of outcomes, and bias in selection of the reported result. Each domain consisted of 3–8 signaling questions. If the signaling questions in every domain was answered with “No/Probably No”, the study was considered to be at low risk of bias and no further signaling questions were considered. If one or more questions were answered by “Yes/Probably Yes”, there was a potential marker for a risk of bias and further questions related to this domain were assessed. Any disagreement was resolved through discussion. The scores are reported in Supplementary Materials Figure S2. Finally, data about the different diagnostic approaches were collected from each study and extracted into a Microsoft Excel spreadsheet.

## 3. Results

### 3.1. Systematic Literature Search

**Figure 1:**
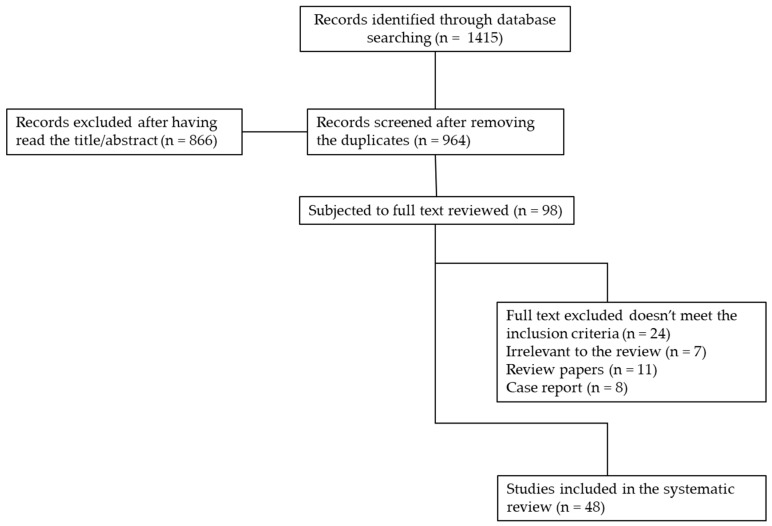
Flow diagram of the results of the systematic search of the literature. Adapted from Moher et al. (2009) and preferred reporting items for systematic reviews and meta-analyses (PRISMA) [26].

The searches yielded 1415 records from the three databases (Figure 1). The articles were all written in English, except for two articles in Chinese that had an English abstract that later led to exclusion. 

### 3.2. Selection of Literature

The 964 articles remaining after duplication removal were screened by title or abstract. This led to 98 records being selected for full text screening. The full text screening exercise led to 24 papers being excluded as they did not meet the inclusion criteria, 7 were found to be irrelevant to the review, 11 were review papers and 8 case reports were not included in the review. In total, 48 papers were subjected to this systematic review. 

## 4. Results of the Literature Search

From the 48 papers reviewed, different diagnostic approaches were considered, as discussed below and summarised in Table 2.

### 4.1. X-ray Radiopaque Markers

**Figure 2:**
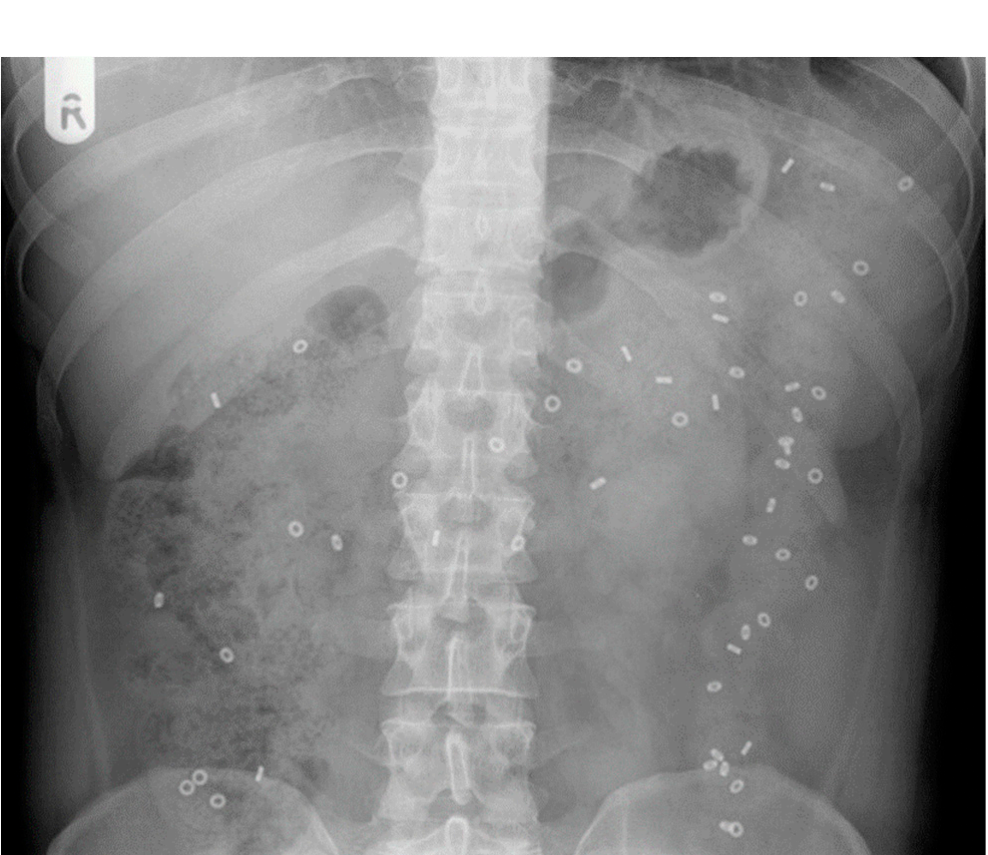
A plain abdominal X-ray image of radiopaque markers (ROMs) in the colon of a patient with constipation.

Traditional approaches include abdominal X-ray. A plain abdominal X-ray can provide important information on transit when combined with tracking ingested markers, such as the radiopaque marker (ROM) method (Figure 2) [27] or barium swallow-based methods [28,29,30].

In the ROM test, one or more capsules containing (~20–50) plastic markers are ingested. The plastic markers contain barium salts, which provide X-ray contrast and make them visible in X-rays. An abdominal radiograph is performed at set time points over the subsequent days. The capsules dissolve and individual markers travel through the upper gastrointestinal tract, enter the colon and gradually move through it. Quantification of whole gut transit time is subsequently performed using simple calculations that assume a steady state loading of the bowel and assign a set amount of time for each marker detected in the gut at the time of imaging [31]. The X-ray film can also be divided anatomically using straight lines to assess segmental colonic transit, for example dividing the distal bowel into right colon, left colon and rectosigmoid [32].

The original method of Metcalf [31] used ingestion of 20 ROM markers each day for 3 days followed by an X-ray taken on day 4 and one taken on day 7. The method has been used throughout the years, including in children with defecation disorders [33]. Many different variants of the technique have been proposed. One “saturation” method uses 10 ring shaped markers ingested each day for 6 days with the addition of ingestion of 20 markers with a different rod shape on days 4, 5 and 6, followed by an X-ray on day 7 [34]. The authors also noted the importance of maintaining a similar time of the day for both ingestion of the markers and imaging in order for the whole gut transit time (WGTT) calculations to be applied correctly. A similar method requiring ingestion only of 10 markers each day for 6 days followed by an X-ray on day 7 was also used [32]. Other methods involved ingestion of a single dose of 20 ROMs and repeated daily X-ray until all markers were evacuated, but not continued if markers were still detected after 7 days [35]. The type of markers used also varies substantially, including rings, rods and also barium balls [36].

In a simple variant of the test, the retention of 20% or more of the markers 5 days after administration is considered indicative of slow transit constipation [37].

Different populations may have shorter average transit times and require an adaptation of the marker methods shown in Western populations. For example, in a study of functional constipation in Indian patients, the participants were asked to ingest a capsule containing 5 markers at time 0, 12 and 24 h followed by X-ray at 36 h after the first ingestion, showing a colon transit time much shorter than in the Western populations [38].

Another version of this technique involves the use of multiple capsules with markers of different geometric shapes. The capsules with markers are taken daily for three days, and abdominal X-rays are performed once or twice (usually 4 days and 7 days after administration). The use of differently shaped markers (usually various types of rings, such as O-rings, D-rings and tri-rings) allows one to visualise the daily movements of the markers through the gastrointestinal tract more precisely. The approach is used for both routine measurements of gastrointestinal transit time.

A barium enema followed by an X-ray film 24 h later has also been used to study colonic transit in children with constipation [28,29]. The method is more invasive, as it involves enema procedures and does not correlate strongly with an X-ray ROM test, with Spearman’s r being in the order of 0.4. A small amount of barium delivered orally was also together with X-ray to measure the transit of a material more similar to chyme than nondigestible objects, such as ROMs [30]. The authors concluded that the small oral barium transit was slower than the ROMs and that it might provide a more reliable method, though it did not attract many citations. 

Despite the variations in type and amount of markers ingested, the ROM method is simple and has been popular for over 4 decades. It is often used as the “gold standard” to validate new methods. For example, ROM whole gut transit tests were used to compare against the new wireless motility capsule (SmartPill^®^) in patients with chronic constipation and in healthy volunteers [39,40]. This wireless capsule measures transit not by imaging, but indirectly from changes in luminal pH [41]. The X-ray images are two-dimensional projections with poor definition of the anatomy, which may create problems in correctly identifying the specific location of markers within the GI tract. The test requires a high level of compliance from patients (i.e., the marker capsules should be taken as instructed). The radiation exposure due to multiple X-rays further limits the usefulness of these techniques, particularly in investigating the causes of constipation in children and pregnant women. Another limitation of the X-ray ROM method is that when two-dimensional radiographs are taken, loops of the bowel can overlap, and therefore the markers located in one part of the bowel can be mistaken for being located in a different segment. 

### 4.2. Gamma Scintigraphy

**Figure 3:**
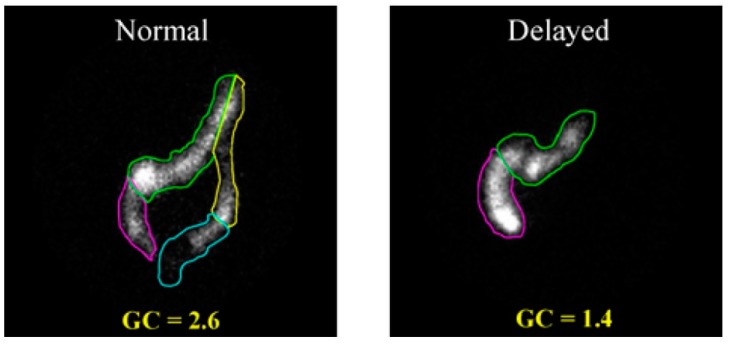
Colonic transit study images acquired with gamma scintigraphy. Reproduced with permission from [61]; published by Springer, 1979.

Gamma scintigraphy is another technique that has long been used to study colonic transit [42,43], including within paediatric population, [44]. The method uses a γ-emitting radioactive isotope. Different isotopes have been used, including ^99^Tc [45], ^111^In [46,47] or ^67^Ga [48,49,50]. The isotopes are administered in a nondigestible or digestible capsule. Subsequent gamma-scintigraphy images (Figure 3) are then obtained at several time points using a large-field-of-view gamma camera. 

Comparing images allows one to establish the speed of movement of the radioactive label through the colon. A variety of methods to deliver the isotopes orally have been proposed. These include a water drink [51,52], a cellulose preparation [53,54], activated carbon [45], minicontainers [55], capsules [52,56,57] and polystyrene pellets [58].

Physiological differences and variation in motor function between individuals can be reflected in variation in the measurement, which otherwise has good reproducibility [59]. The scintigraphic transit time studies can provide the endpoints to evaluate experimental therapies [60]. The technique requires specialist equipment and access to fairly short-lived radioactive isotopes, and thus is limited to a relatively small number of specialised institutions. The use of radioactive materials is an obvious disadvantage, due to the effects of ionising radiation. For this reason, the technique is not well suited for diagnostic investigations of children and pregnant women. 

Scintigraphy transit tests were also used to compare against the new wireless motility capsule (SmartPill^®^) [62].

### 4.3. Tracking Systems

An alternative approach involves the use of magnetic or wireless capsules as the markers of transit.

In the magnetic tracking system approach [63,64], a small magnet is ingested, and its movement through the GI system is tracked using one or more external superconducting quantum interference devices (SQUIDs), which are very sensitive magnetic sensors. The technique allows reliable measurements of gastric, colonic and total transit time without exposure to radiation. The high resolution of magnetic tracking systems allow the study of GI transit in patients with good detail [65]. The specific details of the colonic propulsive dynamics have also been investigated using magnetic tracking [66]. The magnetic tracking system requires magnetic field sensors that are not common, and so far the method remains mostly a research tool.

**Figure 4:**
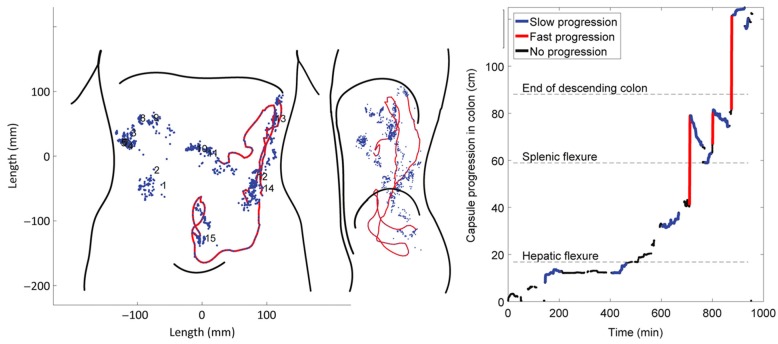
Magnetic tracking system with a wireless telemetric capsule showing the record of space–time representation of capsule activity through the colon. Reproduced with permission from [70]; published by John Wiley & Sons, 1994.

A more advanced magnetic tracking system (Figure 4) uses a wireless telemetric capsule (diameter, 8 mm; length, 21 mm) incorporating a battery and an electromagnet. Following activation, the capsule constantly emits electromagnetic signals that are detected and analysed by a specialised 3D transit system and software [67,68,69,70]. This method has the advantage of being fully ambulatory, as it uses a body-borne detection system. In addition to transit, colorectal length has also been measured using this system [69]. 

Magnetic marker monitoring systems can be used for studying the transit through the GI tract as well as for establishing the fate of oral dosage forms inside it. This is important for targeting delivery of drugs to specific parts of the gastrointestinal tract, including the colon. Small particles of ferromagnetic iron oxide magnetite (Fe_3_O_4_) can be incorporated into the solid dosage forms, and their transit through the GI tract can be monitored using the magnetic tracking system. The net magnetic moment of the particles within the solid form generates a dipole field easily detectable by the system. Disintegration of the dosage leads to the loss of this dipole, as the magnetic particles become reoriented. The timing and anatomic location of this transition points to the position of the GI tract, where the drug becomes released from the solid form [65]. As this method has high spatial (few millimeters) and temporal (milliseconds) resolution, it allows for continuous monitoring of transit, and the release of active pharmaceuticals from the extended release dosage forms can also be studied in detail.

### 4.4. Magnetic Resonance Imaging

Magnetic resonance imaging (MRI) provides a number of significant advantages over X-ray and gamma scintigraphy, such as the lack of radiation exposure and an excellent soft tissue contrast. The MRI methods are noninvasive and typically are not associated with health hazards, except for the classic contraindications for MRI, such as having certain metal implants in the body. The cost of MRI investigation is often cited as one of its major limitations. However, with the development and wider availability of MRI scanners, the costs tend to decrease. The modern instruments allow fast acquisition of information, sometimes with a single breath-hold being sufficient to cover the whole abdomen. This helps to avoid motion artefacts encountered when using longer acquisition times. High-resolution and multiplanar capability are other advantages of this technique. In addition, the lack of ionising radiation is a further advantage when dealing with more vulnerable patients, such as pregnant women or children. The use of other invasive approaches can also be problematic in the geriatric population. 

**Figure 5:**
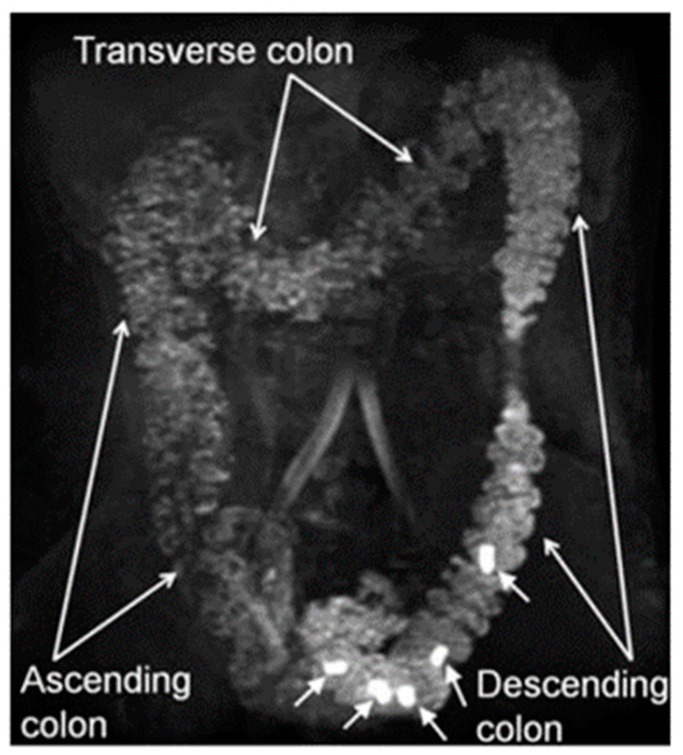
Magnetic resonance imaging (MRI) showing five MRI marker capsules in the colon (indicated by close arrows). Reproduced with permission from [71]; published by John Wiley & Sons, 1994.

Whole gut transit time can be measured with MRI using capsule markers filled with water or a contrast agent, such as gadolinium (Figure 5). MRI does not image directly the outline of the capsules’ shell but images the filling of the capsule. Measurements taken using these markers showed good correlation with the results obtained by other methods. MRI of the whole gut transit time measured using marker capsules (20 mm × 7 mm dimensions) filled with water doped with a gadolinium-based contrast agent were compared against the radiopaque markers technique [71,72]. The capsules were imaged once at 24 h after ingestion to determine their weighted average position score (WAPS) in the gut. The correlation coefficient r between two sets of measurements was 0.85, demonstrating that two techniques are highly consistent and provide similar outcomes. 

The MRI capsules method was applied in studies of adult constipation, whereby whole gut transit measured by MRI was significantly slower for patients with functional constipation compared with healthy controls (*p* < 0.01) [73], and for irritable bowel syndrome with constipation compared with healthy controls (*p* < 0.01 [73] and *p* < 0.03 [74]). Similarly, in another study healthy volunteers swallowed 5 MRI marker capsules (overall outer dimensions 23.9 mm × 6.0 mm) containing a saline water solution doped with gadolinium contrast agent [75]. The capsules were imaged successfully in the GI tract of healthy volunteers at 7 different time points up to 60 h after ingestion to determine their distribution in the GI tract. Another study measured the whole gut transit time in two groups of participants, one with normal gut function and one with slow transit constipation [76]. Both radiopaque markers and gadolinium-based cylindrical marker capsules (15 mm × 0.5 mm dimensions) were used. The correlation coefficients between these two methods were 0.88–0.89 for both groups of participants, suggesting again that MRI can provide a good estimate of gut transit time in both healthy participants and patients with constipation.

Direct oral ingestion of a single-dose, concentrated small drink bolus of gadolinium contrast agent was used in three healthy volunteers with an unprepared bowel [77]. The progress of the bolus though the gastrointestinal system was imaged at 12 h intervals for up to 96 h.

**Figure 6:**
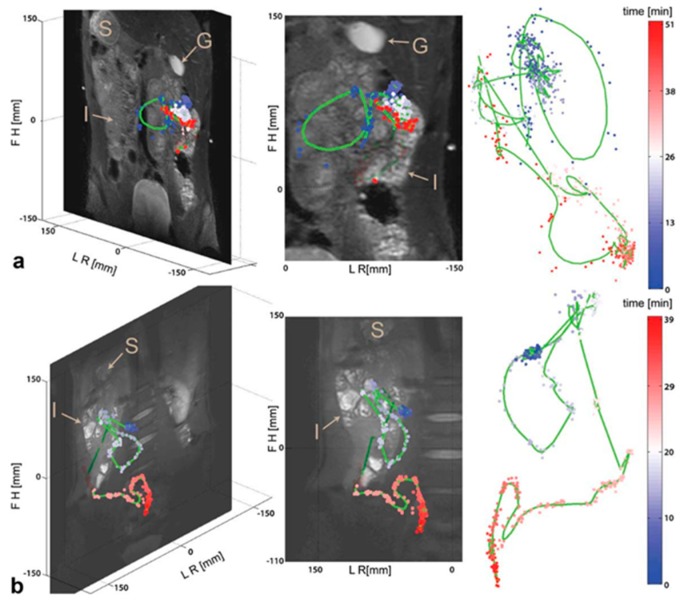
Anatomical reference images of 19F capsule positions and the fitted intestinal course for (**a**) subject A and (**b**) subject B. Stomach (S), gall bladder (G) and small intestine (I) are denoted in the figure. The color code reflects the time course of the two capsules. Reproduced with permission from [78]; published by John Wiley & Sons, 1984.

An interesting alternative to standard water proton MRI imaging of intestinal transit is the tracking of ^19^F-fluorine-labelled capsules (11.5 mm × 7.2 mm dimensions) (Figure 6). Fluorine-based imaging methods provide relatively good sensitivity compared with the more common methods based on the detection of the hydrogen proton signal (^1^H). Taking into account that natural occurrence of fluorine in the human body is close to zero, the detection of the fluorine signal allows for positive detection and contrast. In one study [78], the movement of fluorine-containing capsules ingested with a meal was monitored in participants over extended time periods (up to 1–1.5 h) while they remained in a supine position inside the scanner. This approach, in combination with high resolution anatomical reference scans, allowed accurate visualisation of the capsules’ journey inside the lumen. The ^19^F MRI, however, requires dedicated receiver coils and multinuclear transmitter and receiver hardware, which are not common and mostly based in a few research laboratories. 

An interesting Single-photon Emission Computed Tomography/ Magnetic Resonance SPECT/MR study followed the transit through the gut of a capsule containing both a ^153^Sm label for SPECT and a Gd-doped water solution for MRI, which allowed fused imaging of the two methodologies [79].

An additional advantage of MRI is that a number of parameters of GI motor function can be derived from a single MRI study of a patient, some of which would otherwise require separate techniques and appointments. In addition to GI transit time, MRI can visualise anatomy, measure colonic volume, colonic wall movement (motility), freely mobile water content, chyme relaxometry, gas content and abnormalities of colonic wall thickness [73,74,80,81]. Recently, the MRI tagging technique helped to visualise the movement of colonic chyme in subjects with and without constipation [12]. These new MRI methods have been developed in recent years. There are, however, still limitations associated with them and barriers to adoption in the clinical arena, and the need for further validation and harmonisation of methods across sites remains.

## 5. Conclusions

Identification and treatment of the FGIDs remains a challenging task for practitioners. These functional diseases can be a manifestation of multiple underlying conditions, often with very similar symptoms. The development of novel imaging techniques in recent decades has provided the opportunity to provide objective measures of function, such as gut transit, which could help diagnosis and management. 

The methods and contemporary literature on imaging gastrointestinal transit were briefly reviewed here. More traditional X-ray and gamma scintigraphy methods are still very valuable, and their advantages and limitations were described previously (for example [2,82,83,84]). There is an unmet need for a simple and widely applicable method to provide objective measurements of gastrointestinal transit time in young patients without exposing them to ionising radiation.

New MRI methods are emerging as promising tools to study gastrointestinal transit in FGIDs. We can expect most of the future GI transit imaging developments to come from MRI. The technological challenges of automated methods of data processing will be overcome with improved algorithms and machine learning. This will remove the issues of the use of semiautomated and manual approaches relying on the availability and skills of highly trained staff. Commercial products will enable the translation of these MRI techniques, also addressing the issue of standardisation.

## Figures and Tables

**Table 1:** Patients, intervention, comparator, outcomes, and study design (PICOS).

Parameter	Inclusion Criteria	Exclusion Criteria
Participants	Children and adults of any age with functional gastrointestinal disease of the large bowel. Healthy children and adults of any age who participated in a study aimed at developing a technology or methods to measure whole gut transit.	Animal or in vitro studies.
Intervention	Imaging methods to measure whole gut transit time, particularly new advances in methods.	Studies of effects of bowel cleansing or of a specific drug or nutritional supplement (e.g., fiber) or biofeedback/electrical stimulation and intervention affecting physiology and transit. Direct comparisons of older imaging methods to measure whole gut transit time against each other (e.g., manometry, ultrasound, and X-ray appearance). Pure development of algorithms to calculate whole gut transit time. Epidemiology, prevalence studies. Studies of specific sub-populations (gender, age or race). Proctographic studies. Subcategorisation of patient groups based on transit measurement. Studies of patient reported outcomes scoring systems, symptoms, pain, stools characteristics, colonic gas, gene phenotyping, autoimmune disease, cells, metabolism (e.g., 5HT), molecular biology, transporter and serotonin effects.
Comparator	Not applicable	Not applicable
Outcomes	Imaging methods to measure whole gut transit.	Not applicable
Study design	Randomised controlled trials Quasi-experimental studies (nonrandomised controlled trials, before-and-after, interrupted time series) Observational studies (prospective and retrospective). Studies published in peer-review journals or in the grey literature.	Case reports, reviews or systematic literature reviews and qualitative studies, opinion pieces, editorials, Comments, news, and letters.

**Table 2:** Diagnostic approaches comparison. Note: MRI = magnetic resonance imaging.

Diagnostic Approaches	Advantages	Disadvantages
X-ray Radiopaque Markers	Noninvasive Easily performed test Inexpensive	Ionising radiation exposure Two-dimensional radiographs where loops of the bowel can overlap and segments can be difficult to distinguish, therefore the location of the markers can be difficult to assign Lack of standardisation across centers
Gamma scintigraphy	A well validated method that provides accurate quantitative data for colonic transit Noninvasive, relatively rapid test over 48 to 72 h to evaluate colonic transit	Ionising radiation exposure Multiple image acquisition over consecutive days Limited availability of equipment Costs Radioactive materials (short-lived radioactive isotopes)
Tracking systems	No ionising radiation Fully ambulatory as it uses a body-borne detection system Colorectal length can be measured High spatial and temporal resolution Continuous monitoring of transit	Large sized capsule, may be difficult to swallow Possibility of capsule retention, especially in young children
Magnetic Resonance Imaging	No ionising radiation Excellent soft tissue contrast and image resolution Short scan times Diffusion of equipment worldwide	Contraindication for MRI (e.g., metal implants in the body) Cost

## Supplementary Materials

The following are available online at https://www.mdpi.com/2075-4418/9/4/221/s1.
