# Supplementary material for: Imaging Measurement of Whole Gut Transit Time in Paediatric and Adult Functional Gastrointestinal Disorders: A Systematic Review and Narrative Synthesis
Source: Diagnostics (Basel). 2019 Dec 13;9(4):221. doi: 10.3390/diagnostics9040221 (PMC6963386; doi:10.3390/diagnostics9040221)
Supplement: Supplementary file 1 [file diagnostics-09-00221-s001.pdf]

MEDLINE

1	Gastrointe	4744	Advanced	Display Results	
				More	
2	(transit adj2 (colon* or bowel or gasto* or intestin* or GI)).ti,ab.	4796	Advanced	Display	Results
					More
3	1 or 2	7642	Advanced	Display	Results
					More
4	exp Diagnostic Imaging/	2517281	Advanced	Display	Results
					More
5	(imaging or MRI or "magnetic resonance" or "MR imaging" or "radio-opaque" or radiopaque or ROM or "gamma scintigraphy" or "nuclear medicine" or radiolabel or "radio label" or "wireless telemetric" or "wireless capsule" or "magnetic tracking").ti,ab.	903430	Advanced	Display	Results
					More
6	4 or 5	2883325	Advanced	Display	Results
					More
7	exp Intestinal Diseases/	602406	Advanced	Display	Results
					More
8	("functional gastrointestinal disorder*" or constipat* or "irritable bowel" or IBS or dyspepsia).ti,ab.	44061	Advanced	Display	Results
					More
9	7 or 8	631051	Advanced	Display	Results
					More
10	3 and 6 and 9	701	Advanced	Display	Results
					More
11	limit 10 to (english language and humans)	536	Advanced	Display	Results
					More
12	limit 11 to yr="2014 -Current"	71	Advanced	Display	Results
					More
13	limit 12 to last 10 years	71	Advanced	Display	Results
					More
14	limit 11 to last 10 years	182	Advanced	Display	Results
					More

WoS

# 5	357	(#4) AND I	Edit
		Indexes=SCI-EXPANDED, SSCI, A&HCI, CPCI-S, CPCI-SSH, BKCI-S, BKCI-SSH, ESCI, CCR-EXPANDED, IC Timespan=All years	
# 4	371	#3 AND #2	Edit
		Indexes=SCI-EXPANDED, SSCI, A&HCI, CPCI-S, CPCI-SSH, BKCI-S, BKCI-SSH, ESCI, CCR-EXPANDED, IC Timespan=All years	
# 3	57,114	TS=("func	Edit
		Indexes=SCI-EXPANDED, SSCI, A&HCI, CPCI-S, CPCI-SSH, BKCI-S, BKCI-SSH, ESCI, CCR-EXPANDED, IC Timespan=All years	
# 2	2,538,059	TS=(imagi	Edit
		Indexes=SCI-EXPANDED, SSCI, A&HCI, CPCI-S, CPCI-SSH, BKCI-S, BKCI-SSH, ESCI, CCR-EXPANDED, IC Timespan=All years	
# 1	7,617	TS=(transi	Edit
		Indexes=SCI-EXPANDED, SSCI, A&HCI, CPCI-S, CPCI-SSH, BKCI-S, BKCI-SSH, ESCI, CCR-EXPANDED, IC Timespan=All years	

Supplementary Figure 1: Databases search strategy.

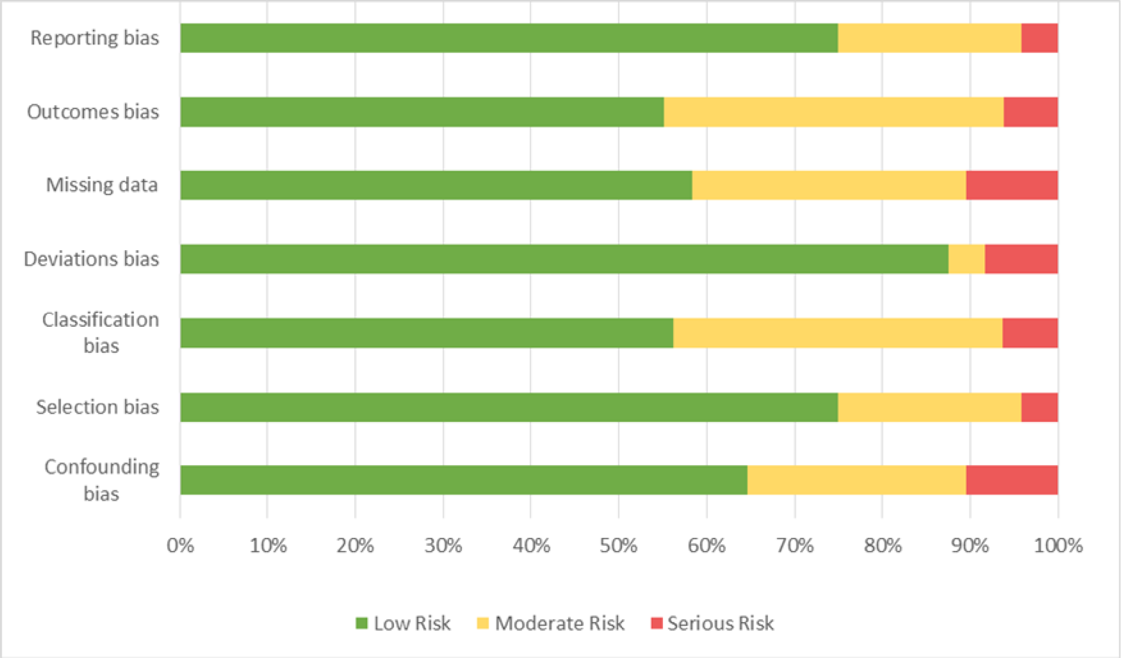

Supplementary Figure 2: Quality scores for the risk of bias using the ROBINS tool.
